# Supplementary material for: Galectin-9 suppresses B cell receptor signaling and is regulated by I-branching of N-glycans
Source: Nat Commun. 2018 Aug 17;9:3287. doi: 10.1038/s41467-018-05770-9 (PMC6098069; doi:10.1038/s41467-018-05770-9)
Supplement: Supplementary file 1 — Supplementary Information [file 41467_2018_5770_MOESM1_ESM.pdf]

<u>Supplementary Information</u>

Galectin-9 suppresses B cell receptor signaling and is regulated by I-branching of N-glycans

Giovannone *et al*

Supplementary Figures:

Supplementary Fig. 1: Sort purity assessment of human tonsillar B cell subsets used in N-glycome analysis

Supplementary Fig. 2: MALDI-TOF mass spectra of permethylated N-glycans from isolated human B cells

Supplementary Fig. 3: Plant lectin analysis of human B cell glycosylation

Supplementary Fig. 4: Gal-9 binding analysis to primary tonsillar B cells and validation of *GCNT2* variant B cell lines

Supplementary Fig. 5: Gal-9 is highly expressed by naïve B cells in B cell follicles and binds CD45

Supplementary Fig. 6: Gal-9 inhibits BCR-mediated phosphorylation of NF-κB and JNK but does not influence signaling through canonical BCR-associated molecules

Supplementary Fig. 7: Gal-9 equally inhibits stimulation through IgM- and IgD-BCRs

Supplementary Fig. 8: Gal-9 does not induce substantial internalization of CD45, CD22, or BCR

Supplementary Fig. 9: Full Western blots from this study

Supplementary Tables:

Supplementary Table 1: Primers and shRNAs used in this study

Supplementary Table 2: Reagents used in this study

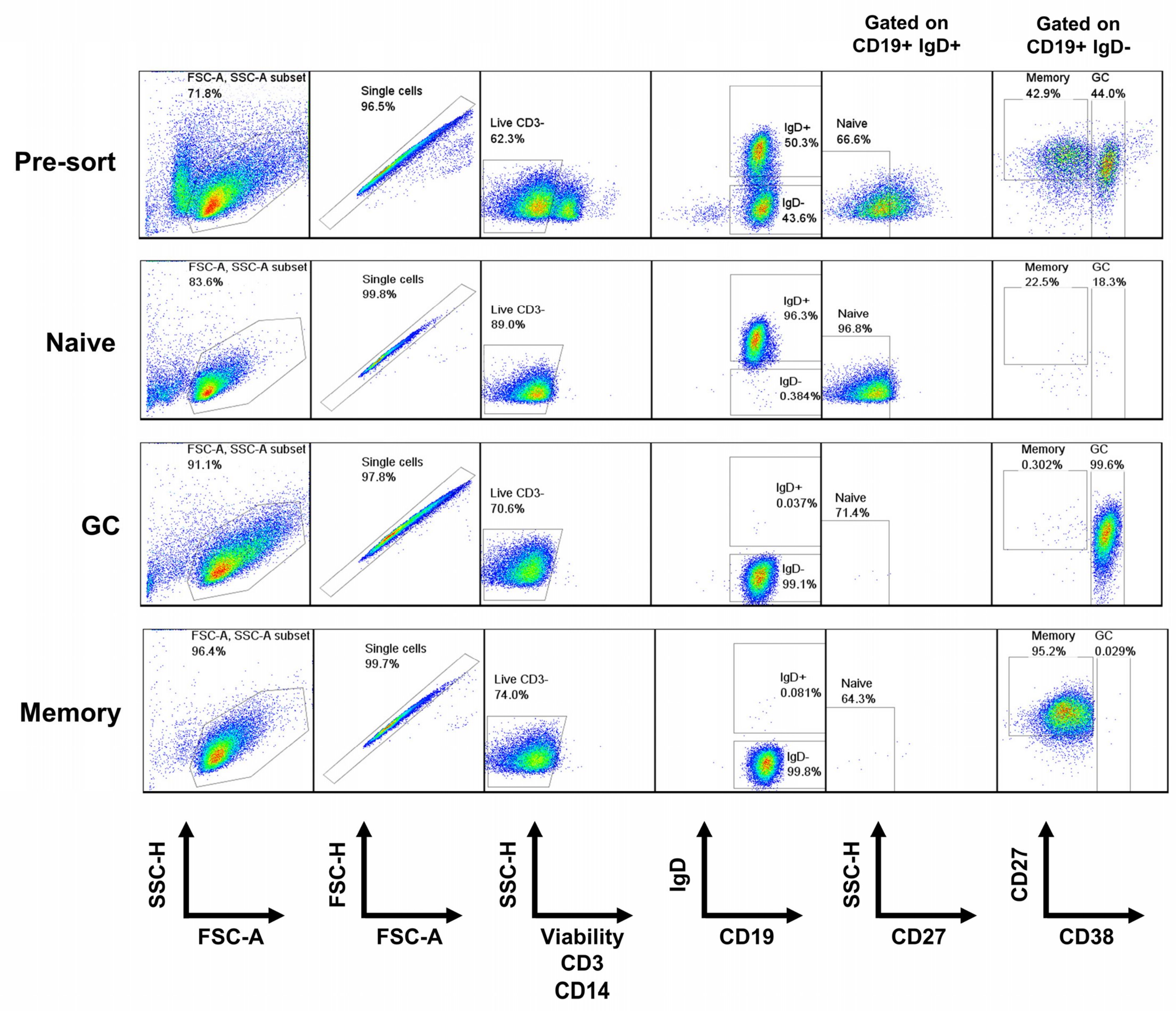

Supplementary Fig. 1: **Sort purity assessment of human tonsillar B cell subsets used in N-glycome analysis**. Pre- and post-sort evaluation of B cell populations subsequently used for MALDI-TOF analysis of N-glycans. To obtain sufficient numbers for N-glycome analysis (>20 million sorted cells), cryopreserved tonsil mononuclear cells from the same tonsil specimen were thawed, sorted, and snap-frozen on five different days. B cell pellets were processed and analyzed together in subsequent N-glycome assessments. Results are representative of all five sorts.

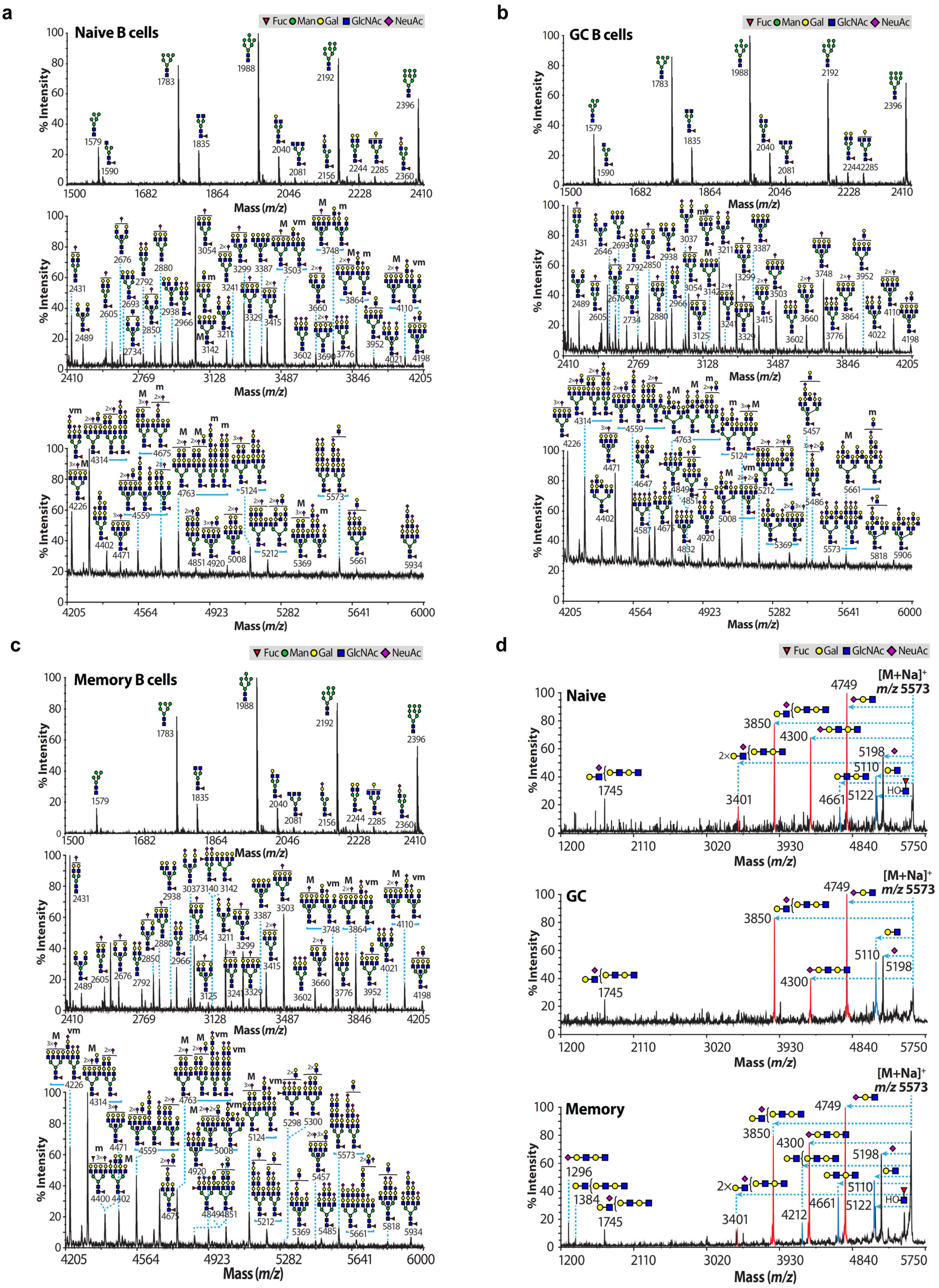
a
Naive B cells
Fuc Man Gal GlcNAc NeuAc
% Intensity
Mass (m/z)
1579
1590
1783
1835
1988
2040
2081
2156
2192
2244
2285
2360
2396
2431
2489
2605
2676
2693
2734
2792
2850
2880
2938
2966
3054
3142
3211
3241
3299
3329
3387
3415
3503
3602
3660
3690
3748
3776
3864
3952
4021
4110
4198
4226
4314
4402
4471
4559
4675
4763
4851
4920
5008
5124
5212
5369
5573
5661
5934
b
GC B cells
c
Memory B cells
d
Naive
GC
Memory
[M+Na]+
m/z 5573
1296
1384
1745
3401
3850
4212
4300
4661
4749
5110
5122
5198
HO

Supplementary Fig. 2: **MALDI-TOF mass spectra of permethylated N-glycans from isolated human B cells**. **(a)** N-glycomic profile of naive; **(b)**, GC and **(c)**, memory B cells sorted by flow cytometry from human tonsil as in **Fig. 1a,b** and **Supplementary Fig. 1**. Profiles of N-glycans are from the 50% MeCN fraction from a C_{18} Sep-Pak. Structures that show sugars outside a bracket have not been unequivocally defined. Structures labeled with **M**, **m,** and **vm** in bold indicate major, minor and very minor abundances respectively. Putative structures based on composition, tandem mass spectrometry and knowledge of biosynthetic pathways. The relative antenna position of each tri- and tetra-antennary structure has not been determined. All molecular ions are $[M+Na]^+$. (**d**) Representative MALDI-TOF-TOF MS/MS spectra of the molecular ion found at *m/z* 5573 on naive, GC, and memory B cells (presented in (a), (b) and (c), respectively). Horizontal dashed lines correspond to indicated losses from the molecular ion $[M+Na]^+$. Solid vertical red and blue lines correspond to the relative ion intensity peaks depicting sialylated and non-sialylated losses from the molecular ion respectively. For naïve and GC B cell spectra, data shown are from one of two experiments, each with a distinct tonsil specimen, with similar findings. Data from memory B cells are from a single experiment.

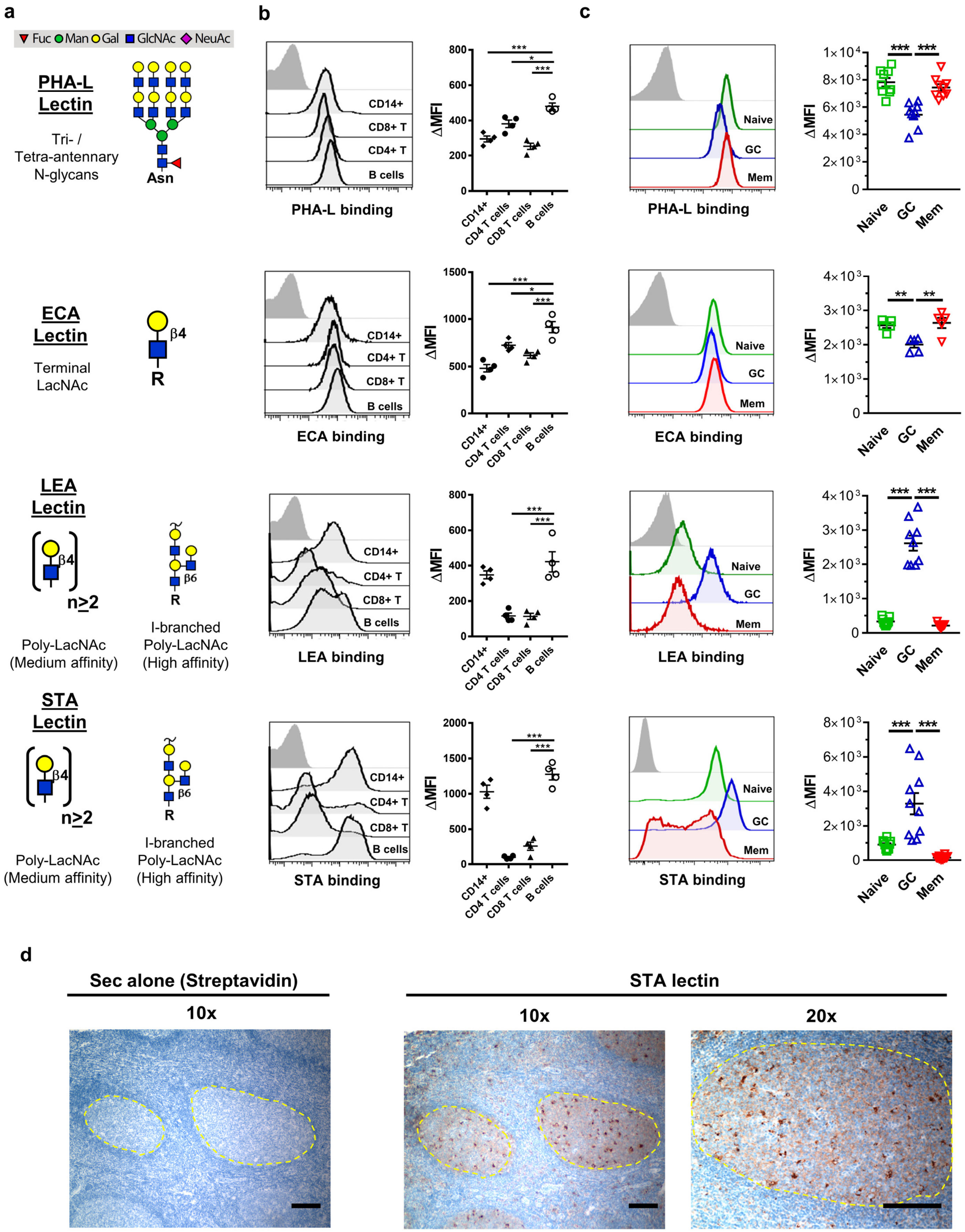
a
b
c
Fuc Man Gal GlcNAc NeuAc
PHA-L Lectin
Tri- / Tetra-antennary N-glycans
Asn
PHA-L binding
CD14+
CD8+ T
CD4+ T
B cells
ΔMFI
CD14+
CD4 T cells
CD8 T cells
B cells
Naive
GC
Mem
ECA Lectin
Terminal LacNAc
β4
R
ECA binding
LEA Lectin
n≥2
β6
Poly-LacNAc (Medium affinity)
I-branched Poly-LacNAc (High affinity)
LEA binding
STA Lectin
STA binding
d
Sec alone (Streptavidin)
STA lectin
10x
10x
20x

Supplementary Fig. 3: Plant lectin analysis of human B cell glycosylation. (a) Preferred glycan binding specificities of plant lectins used in (b) and (c). **(b)** Comparative plant lectin analysis of tonsillar leukocyte glycosylation by flow cytometry. *Left*, representative histograms; *right*, quantified mean fluorescence intensities (MFI). **(c)** Plant lectin analysis of B cell glycosylation by flow cytometry. *Left*, representative histograms; *right*, quantified mean fluorescence intensities (MFI). **(d)** Immunohistochemical staining of formalin-fixed, paraffin-embedded tonsil sections with STA lectin. Yellow dashes delineate GCs within B cell follicles. Scale bar, 200µm. For (b), n=4, where each data point represents a different tonsil specimen pooled from two independent experiments. For (c), n=10 (PHA-L, LEA, STA) or n=5 (ECA), where each data point represents a distinct tonsil specimen, from one (ECA) or pooled from two (PHA-L, LEA, STA) independent experiment. For (d), data are representative of three independent experiments using three different tonsil specimens. For (b) and (c), statistics were calculated using one-way ANOVA with correction for multiple comparisons. Throughout, bars and error bars depict mean and SEM, respectively. ns = not significant, *p≤0.05, **p≤0.01, ***p≤0.001.

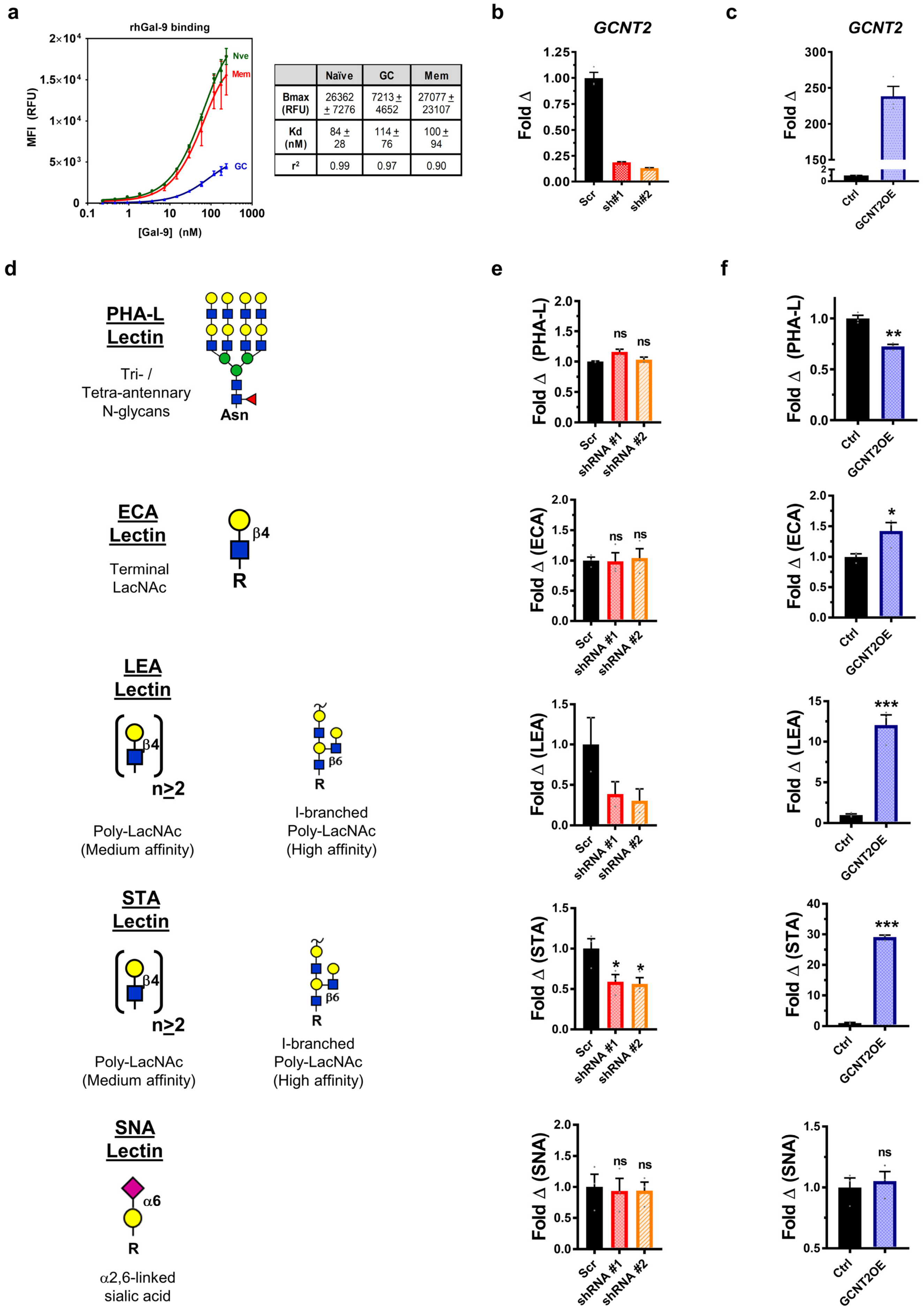

	Naïve	GC	Mem
Bmax (RFU)	26362 ± 7276	7213 ± 4652	27077 ± 23107
Kd (nM)	84 ± 28	114 ± 76	100 ± 94
r^2	0.99	0.97	0.90

Supplementary Fig. 4: Gal-9 binding analysis to primary tonsillar B cells and validation of *GCNT2* variant B cell lines. (a) Saturation binding curve of recombinant Gal-9 binding to naïve, GC, and memory B cells, measured by flow cytometry. Non-linear regression analysis was performed in Prism 7.0 (GraphPad) using the "Total binding, one site" method. B_{max}, K_D, and r^2 values are given with 95% confidence intervals. (b) Validation of lentiviral *GCNT2* shRNA-mediated knockdown in Ramos B cells and (c) *GCNT2* overexpression in NUDUL-1 B cells by qRT-PCR. (d) Preferred glycan binding specificities of plant lectins used in (e) and (f). **(e)** Analysis of glycan alterations following *GCNT2* knockdown and **(f)** *GCNT2* overexpression by flow cytometric staining with plant lectins of defined glycan-binding preference (depicted in (d)). For (a), n=3 tonsil specimens pooled from three independent experiments. For (b) and (c), data are from a single experiment, and error bars represent technical replicates. For (e) and (f), error bars represent biological replicates pooled from three independent experiments. For (e), statistics were calculated using one-way ANOVA with correction for multiple comparisons. For (f), statistics were calculated using an unpaired, two-tailed t-test. Throughout, bars and error bars depict mean and SEM, respectively. ns = not significant, *$p \leq 0.05$, **$p \leq 0.01$, ***$p \leq 0.001$.

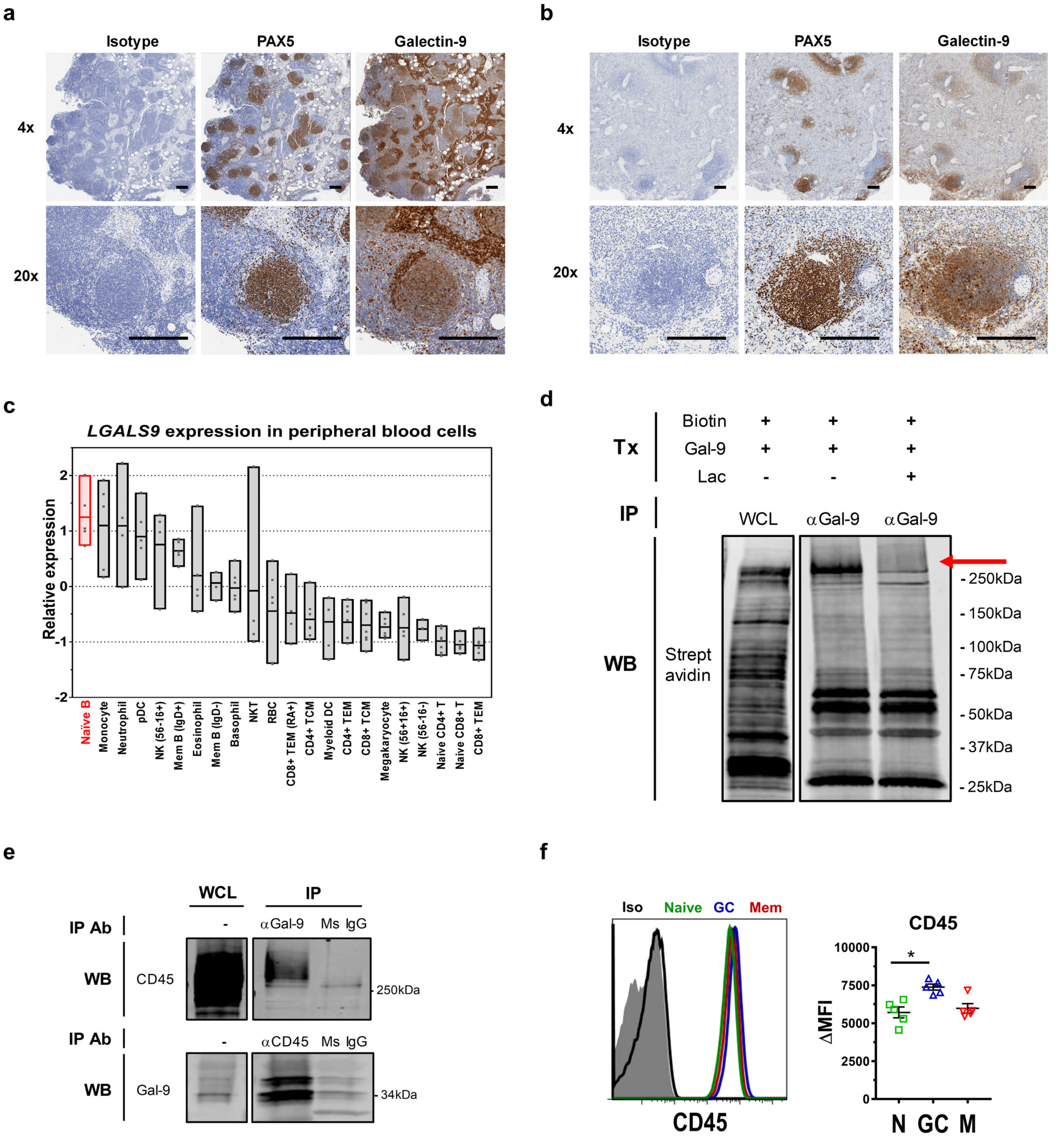
a
Isotype
PAX5
Galectin-9
4x
20x
b
Isotype
PAX5
Galectin-9
4x
20x
c
LGALS9 expression in peripheral blood cells
Relative expression
2
1
0
-1
-2
Naïve B
Monocyte
Neutrophil
pDC
NK (56-16+)
Mem B (IgD+)
Eosinophil
Mem B (IgD-)
Basophil
NKT
RBC
CD8+ TEM (RA+)
CD4+ TCM
Myeloid DC
CD4+ TEM
CD8+ TCM
Megakaryocyte
NK (56+16+)
NK (56-16-)
Naive CD4+ T
Naive CD8+ T
CD8+ TEM
d
Tx
Biotin + + +
Gal-9 + + +
Lac - - +
IP
WCL
αGal-9
αGal-9
WB
Strept avidin
250kDa
150kDa
100kDa
75kDa
50kDa
37kDa
25kDa
e
WCL
IP
IP Ab
-
αGal-9
Ms IgG
WB
CD45
250kDa
IP Ab
-
αCD45
Ms IgG
WB
Gal-9
34kDa
f
Iso
Naive
GC
Mem
CD45
CD45
10000
7500
5000
2500
0
ΔMFI
*
N
GC
M

Supplementary Fig. 5: Gal-9 is highly expressed by naïve B cells in B cell follicles and binds CD45. (a) Immunohistochemical stains of Gal-9, PAX5 (to identify B cells), and isotype control in formalin-fixed, paraffin-embedded sections of human lymph node and **(b)** spleen, counterstained with hematoxylin. Scale bar, 200μm. **(c)** Relative mean *LGALS9* (Gal-9) expression in peripheral blood cells, analyzed from the publicly available dataset, GSE24759 [https://www.ncbi.nlm.nih.gov/geo/query/acc.cgi?acc=GSE24759]. Floating bars depict minimum, mean, and maximum expression values. **(d)** Immunoprecipitation (IP) of glycoproteins associating with Gal-9 in resting B cells. GC-depleted tonsil B cells were magnetically enriched, biotinylated (to label cell surface proteins), labeled with recombinant Gal-9 (2μg mL^{-1}), lysed, and immunoprecipitated with Gal-9 Ab. Immunoprecipitated glycoproteins were detected by Western blot with labeled streptavidin. To control for non-carbohydrate-dependent interactions, cells were incubated with lactose to elute Gal-9 before IP. *Red arrow,* specific band detectable in Gal-9 IP but absent in lactose control. **(e)** Co-IP of endogenous Gal-9 and CD45 in naïve B cells. Untouched magnetically-enriched naïve B cells were lysed and subject to IP with Gal-9, CD45, or matched isotype control Ab. WCL, whole cell lysate. **(f)** Analysis of tonsil B cell CD45 expression by flow cytometry. For (b) and (c), n=3 distinct tonsil specimens from three independent experiments. For (d), and (e), data are representative of three experiments from three distinct tonsil specimens. For (f), n=5, where each data point represents a distinct tonsil specimen. Throughout, bars and error bars depict mean and SEM, respectively. *$p \leq 0.05$.

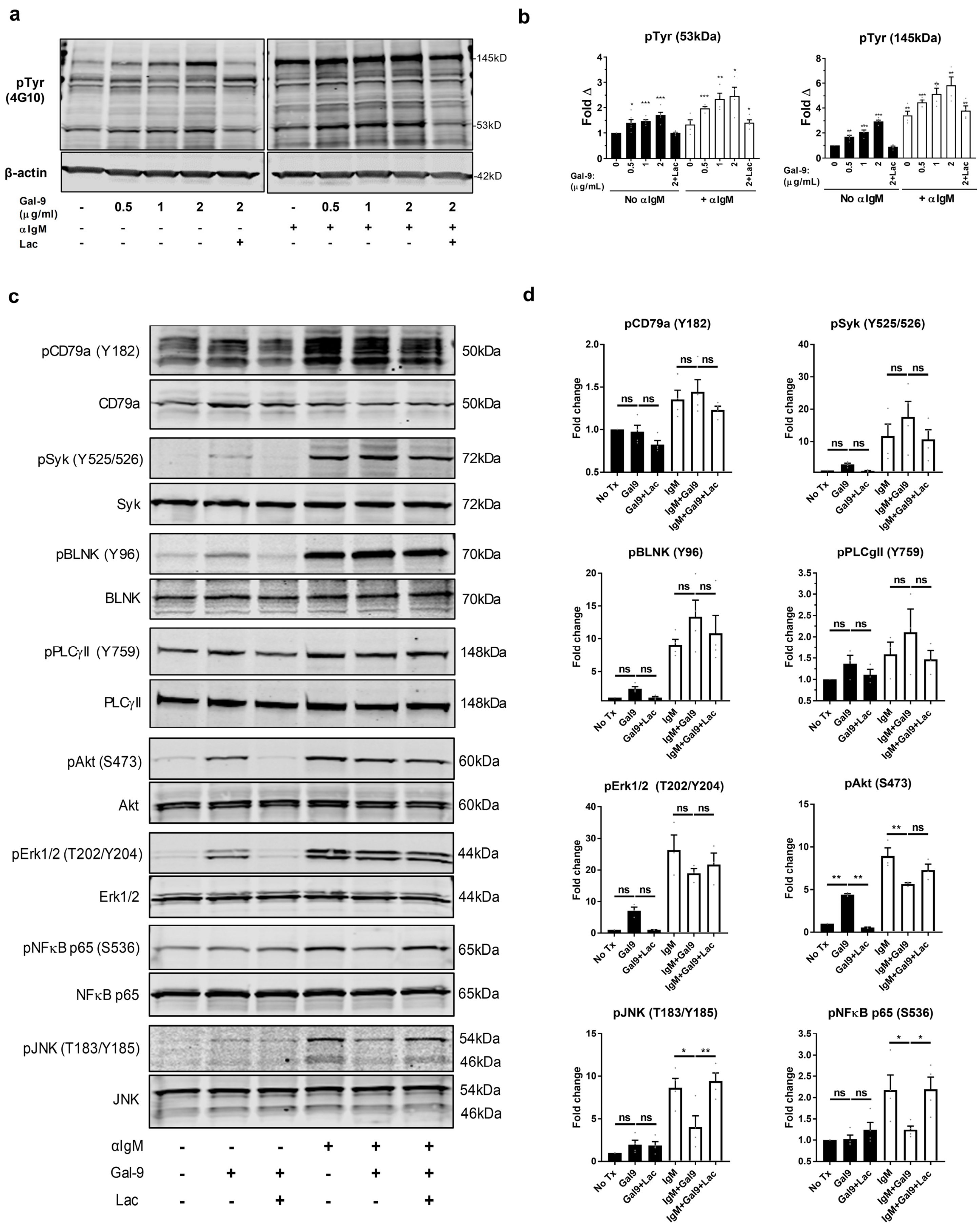
a
pTyr
(4G10)
145kD
53kD
β-actin
42kD
Gal-9
(μg/ml)
- 0.5 1 2 2 - 0.5 1 2 2
αIgM
- - - - - + + + + +
Lac
- - - - + - - - - +
b
pTyr (53kDa)
pTyr (145kDa)
Fold Δ
Gal-9:
(μg/mL)
0 0.5 1 2 2+Lac 0 0.5 1 2 2+Lac
No αIgM
+ αIgM
c
pCD79a (Y182) 50kDa
CD79a 50kDa
pSyk (Y525/526) 72kDa
Syk 72kDa
pBLNK (Y96) 70kDa
BLNK 70kDa
pPLCγII (Y759) 148kDa
PLCγII 148kDa
pAkt (S473) 60kDa
Akt 60kDa
pErk1/2 (T202/Y204) 44kDa
Erk1/2 44kDa
pNFκB p65 (S536) 65kDa
NFκB p65 65kDa
pJNK (T183/Y185) 54kDa 46kDa
JNK 54kDa 46kDa
αIgM - - - + + +
Gal-9 - + + - + +
Lac - - + - - +
d
pCD79a (Y182)
pSyk (Y525/526)
pBLNK (Y96)
pPLCgII (Y759)
pErk1/2 (T202/Y204)
pAkt (S473)
pJNK (T183/Y185)
pNFκB p65 (S536)
Fold change
No Tx
Gal9
Gal9+Lac
IgM
IgM+Gal9
IgM+Gal9+Lac
ns
*
**

Supplementary Fig. 6: Gal-9 inhibits BCR-mediated phosphorylation of NF-κB and JNK but not canonical BCR-associated molecules. (a) Representative Western blot and **(b)** quantification of tyrosine phosphorylation (4G10 mAb) for the indicated bands following magnetic enrichment of naïve B cells by negative selection and treatment with or without anti-IgM crosslinking antibody (15μg mL^{-1}) and/or Gal-9 (as indicated) for 5min. Where noted, 25mM lactose was included as a negative control for Gal-9 carbohydrate-binding activity. Phospho-tyrosine signal was normalized to respective β-actin levels and displayed relative to no treatment condition. "No IgM" and "αIgM" lanes were loaded on the same gel and probed on the same blot. **(c)** Representative Western blot and **(d)** quantification of BCR signaling in magnetically enriched, untouched naïve B cells treated with anti-IgM crosslinking $F(ab')_2$ (15μg mL^{-1}) with or without Gal-9 (2μg mL^{-1}) for 5min (CD79a, Syk, BLNK, PLCγII) or 30mins (Erk, Akt, JNK, NF-κB). Blots were probed for the indicated phospho-proteins, then stripped and re-probed for the respective total protein. Phospho-protein signal was normalized to respective total protein levels. Data are displayed relative to no treatment condition. Where indicated, 25mM lactose was also included. For (a) and (c), data are representative of four independent experiments. For (b), n=4 or more different tonsil specimens pooled from four or more independent experiments, respectively. For (d) n=4 different tonsil specimens pooled from four independent experiments, except PLCγII, where n=3. Statistics were calculated using one-way ANOVA with correction for multiple comparisons. Throughout, bars and error bars depict mean and SEM, respectively. ns = not significant, *$p \leq 0.05$, **$p \leq 0.01$, ***$p \leq 0.001$.

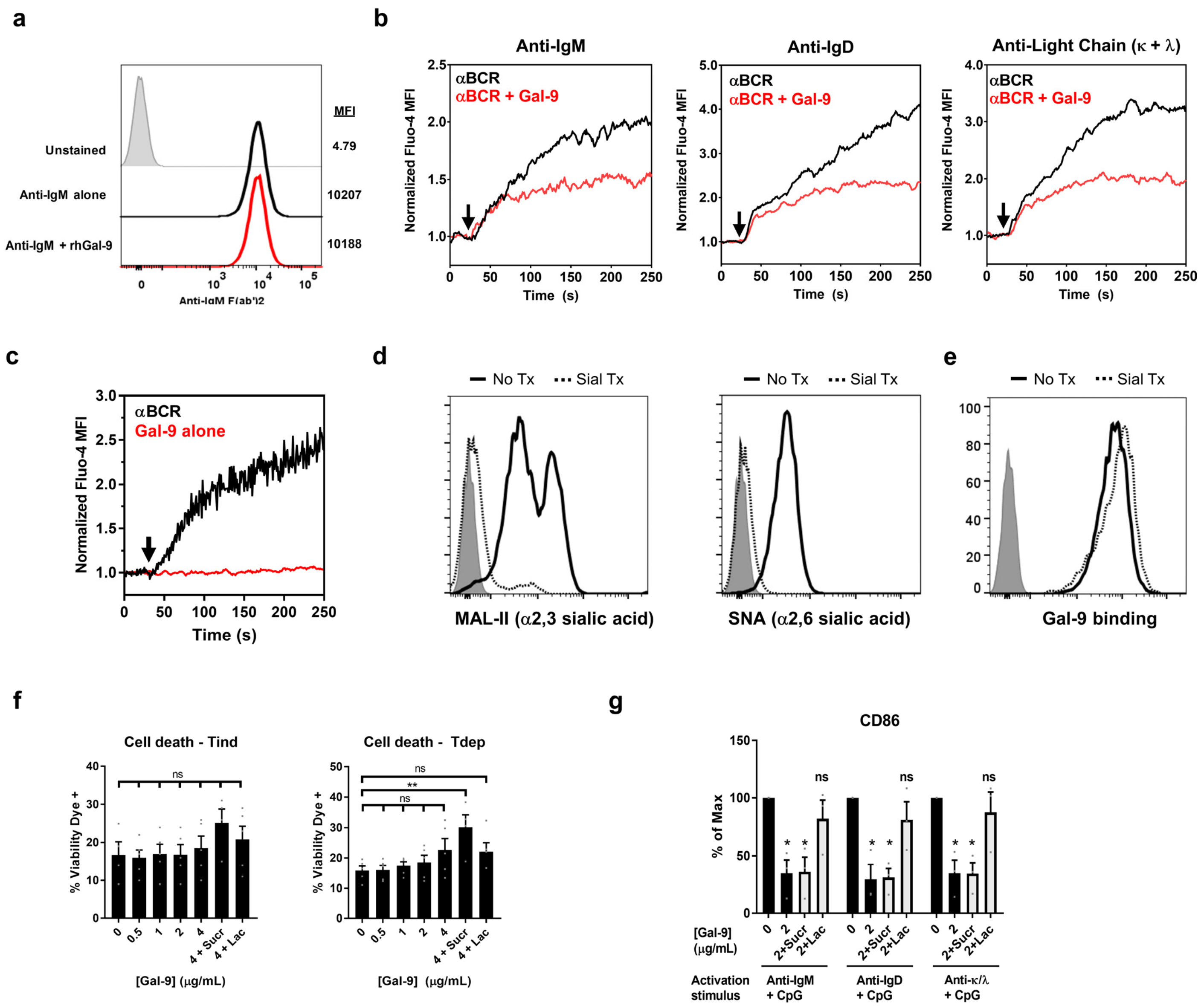
a
b
c
d
e
f
g
MFI
Unstained 4.79
Anti-IgM alone 10207
Anti-IgM + rhGal-9 10188
Anti-IgM F(ab')2
Anti-IgM
Anti-IgD
Anti-Light Chain (κ + λ)
αBCR
αBCR + Gal-9
Normalized Fluo-4 MFI
Time (s)
Gal-9 alone
No Tx
Sial Tx
MAL-II (α2,3 sialic acid)
SNA (α2,6 sialic acid)
Gal-9 binding
Cell death - Tind
Cell death - Tdep
% Viability Dye +
[Gal-9] (μg/mL)
ns
**
CD86
% of Max
Activation stimulus
Anti-IgM + CpG
Anti-IgD + CpG
Anti-κ/λ + CpG

Supplementary Fig. 7: Gal-9 equally inhibits stimulation through IgM- and IgD-BCRs. (a) Comparison of anti-IgM F(ab')$_2$ staining in the presence or absence of recombinant Gal-9. Magnetically-enriched naïve B cells were stained for 15 minutes at room temperature with polyclonal IgM F(ab')$_2$ -AlexaFluor 647 (10μg mL^{-1}) with or without Gal-9 (2.5μg mL^{-1}). **(b)** Comparison of BCR-mediated calcium flux, as in **Fig. 5a**, following stimulation of magnetically enriched, untouched naïve B cells with either anti-human IgM F(ab')$_2$, anti-human IgD F(ab')$_2$, or a combination of anti-human κ F(ab')$_2$ and anti-human λ F(ab')$_2$ (all 20μg mL^{-1}). **(c)** Calcium flux assay of primary B cells treated with anti-human IgM F(ab')$_2$ or Gal-9 alone, as in **Fig. 5a**. **(d)** Analysis of cell surface sialic acids on primary tonsil mononuclear cells following treatment with *Arthrobacter ureafaciens* sialidase (Sial), as measured by staining with two sialic acid-specific plant lectins, *Maackia amurensis* lectin-II (MAL-II) and *Sambucus nigra* agglutinin (SNA). **(e)** Assessment of recombinant Gal-9 binding to IgD$^+$ B cells following treatment with *Arthrobacter ureafaciens* sialidase (Sial). **(f)** Cell viability assessment of data presented in **Fig. 6a** using Zombie Near-infrared (NIR) fixable viability dye (Biolegend). "Lac" indicates 10mM Lactose, "Sucr" indicates 10mM sucrose, a non-inhibitory osmolarity control. **(g)** Comparison of naïve B cell CD86 (B7-2) expression 40hr post-activation with either anti-IgM, anti-IgD, or anti-κ/λ light chain crosslinking F(ab')$_2$ (all 10μg mL^{-1}), plus unmethylated CpG oligonucleotides and IL-2/4/10, as in **Fig. 6c**. "2+Lac" indicates 2μg Gal-9 plus 10mM lactose; "2+Sucr" indicates 2μg Gal-9 plus 10mM sucrose. For (a-c), data depict results from a single experiment. Data in (d) and (e) are representative of three and two independent experiments, respectively. For (f), n=5 separate tonsil specimens pooled from five independent experiments. For (g), n=3 tonsil specimens pooled from three independent experiments. For (f), statistics were calculated using one-way ANOVA corrected for multiple comparisons. For (g), statistics were calculated using a one-sample t-test against a hypothetical value of 100. Throughout, bars and error bars depict mean and SEM, respectively. ns = not significant, $*p \leq 0.05$, $**p \leq 0.01$, $***p \leq 0.001$.

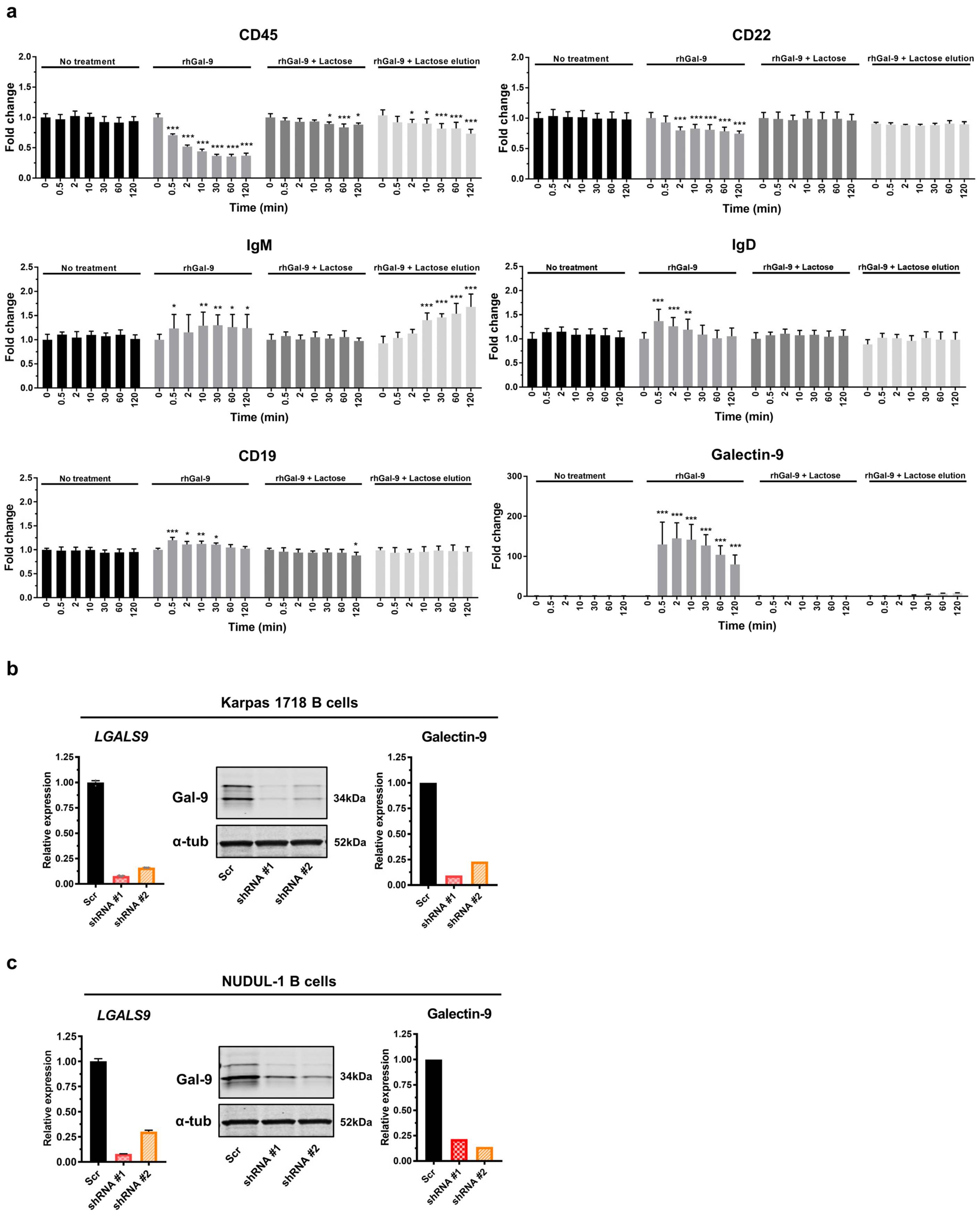
a
CD45
CD22
IgM
IgD
CD19
Galectin-9
No treatment
rhGal-9
rhGal-9 + Lactose
rhGal-9 + Lactose elution
Fold change
Time (min)
0
0.5
2
10
30
60
120
b
Karpas 1718 B cells
LGALS9
Galectin-9
Relative expression
Gal-9
α-tub
34kDa
52kDa
Scr
shRNA #1
shRNA #2
c
NUDUL-1 B cells
LGALS9
Galectin-9
Relative expression
Gal-9
α-tub
34kDa
52kDa
Scr
shRNA #1
shRNA #2

Supplementary Fig. 8: Gal-9 does not induce internalization of CD45, CD22, or BCR. (a) Flow cytometric analysis of glycoprotein internalization on magnetically-enriched, untouched naïve B cells. B cells were treated for the indicated time at 37°C with either no treatment, 2μg mL^{-1} recombinant human Gal-9 (rhGal-9), or 2μg mL^{-1} Gal-9 with 25mM Lactose (rhGal-9 + lactose), before being iced, washed, and stained with monoclonal Abs against the indicated glycoproteins. To ascertain between bona fide internalization and steric blockade of mAb binding by Gal-9, a separate condition was also analyzed wherein cells were incubated with Gal-9 in the absence of lactose, but washed immediately prior to mAb staining with buffer containing 25mM lactose to elute bound Gal-9 (rhGal-9 + lactose elution). **(b)** Validation of *LGALS9* shRNA-mediated knockdown in Karpas 1718 B cells (Gal-9^{hi}, $CD22^{+}$, SHP-1^{+}) by qRT-PCR (left) and Western blot (center, quantified at right). **(c)** Validation of *LGALS9* knockdown in NUDUL-1 B cells (Gal-9^{hi}, $CD22^{lo}$, SHP-1^{lo}) by qRT-PCR (left) and Western blot (center, quantified at right). For (a), n=3 tonsil specimens over three independent experiments, except lactose elution condition, where n=2. Data in (b) and (c) are from a single experiment and error bars represent technical replicates. Statistics in (a) were calculated using one-way ANOVA corrected for multiple comparisons. Throughout, bars and error bars depict mean and SEM, respectively. ns = not significant, $*p\leq0.05$, $**p\leq0.01$, $***p\leq0.001$.

Western blots corresponding with Figure 3 (1 of 2)

Fig. 3c: Tonsil Gal-9 expression

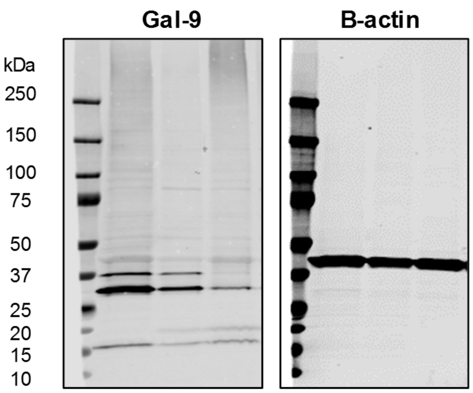

Fig. 3e (Gal-9 IP, CD45 blot; low exposure)

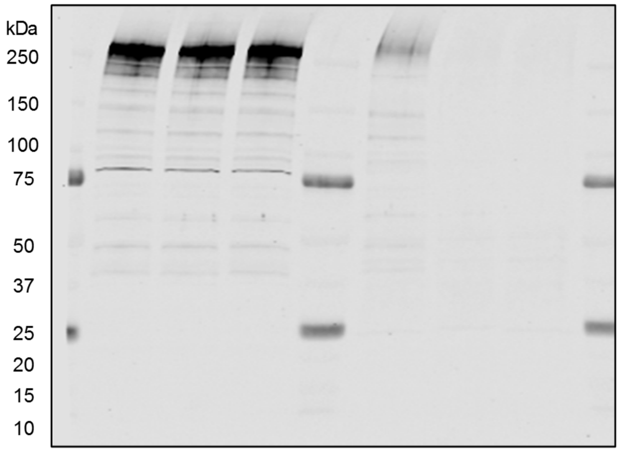

Fig. 3e (CD45 IP, Gal-9 blot)

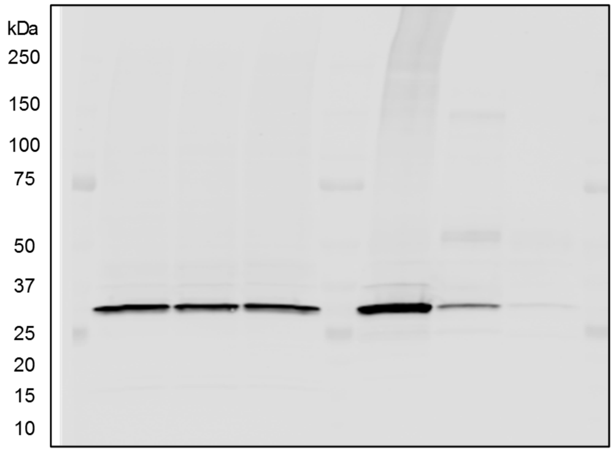

Fig. 3e (Gal-9 IP, CD45 blot; higher exposure)

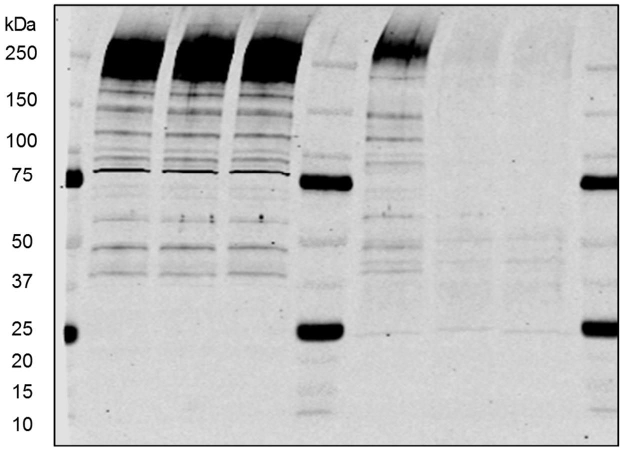

Western blots corresponding with Figure 3 (2 of 2)

Fig. 3e (Gal-9 IP, Gal-9 blot; low exposure)

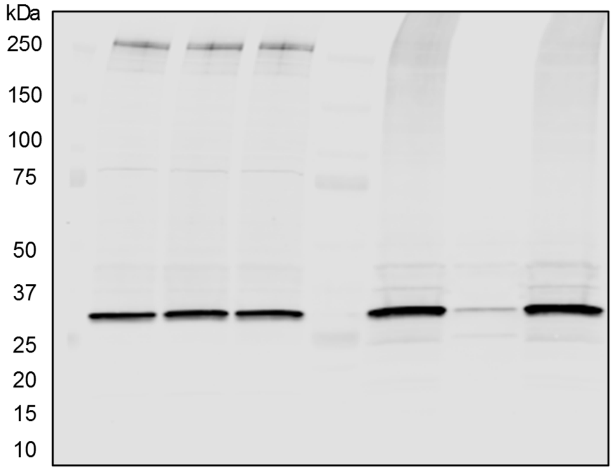

Fig. 3e (CD45 IP, CD45 blot)

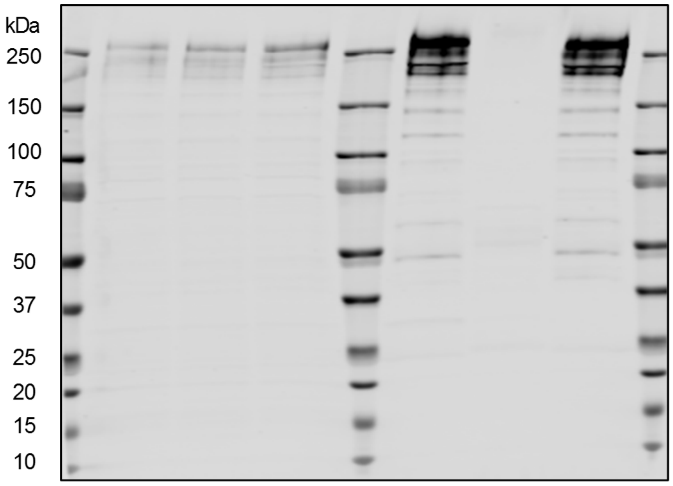

Western blots corresponding with Figure 4

Fig. 4a - pLyn blot (also blotted with pCD22) Higher exposure

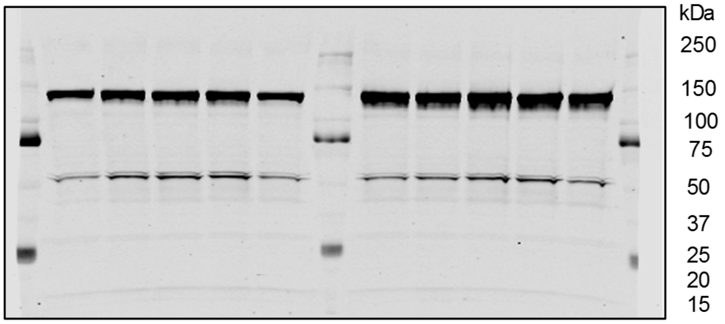

Fig. 4a – total Lyn blot (also blotted with total CD22) Higher exposure

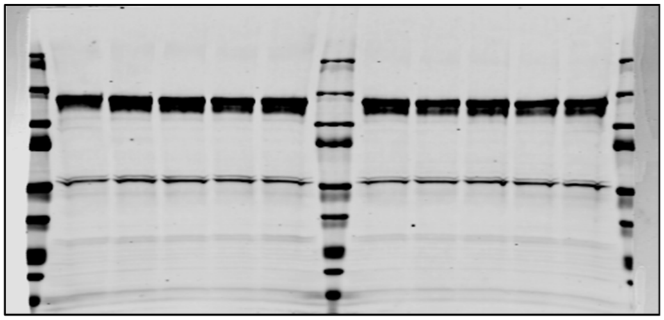

Fig. 4a – pCD22 blot (also blotted with pLyn) Lower exposure

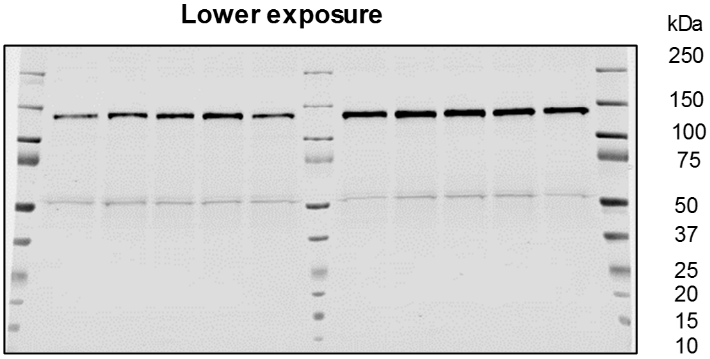

Fig. 4a – total CD22 blot (also blotted with total Lyn) Lower exposure

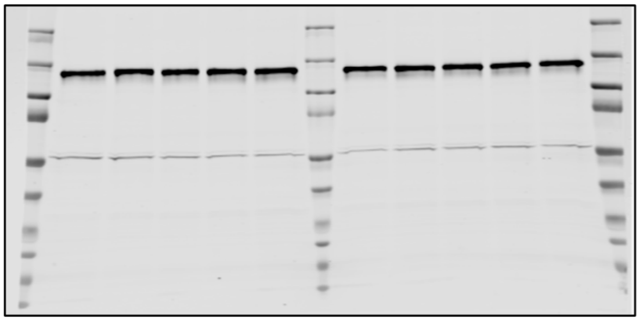

Fig. 4a – pSHP-1 blot

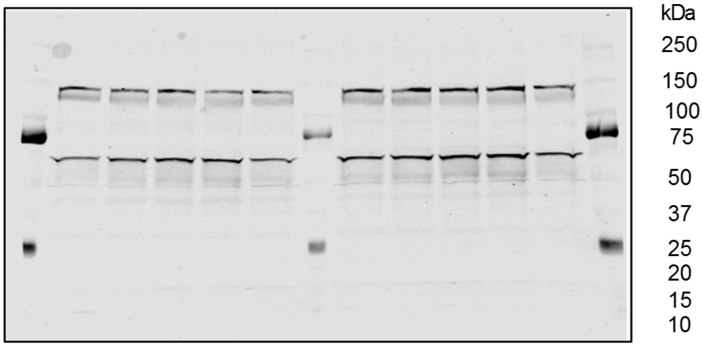

Fig. 4a – total SHP1 blot

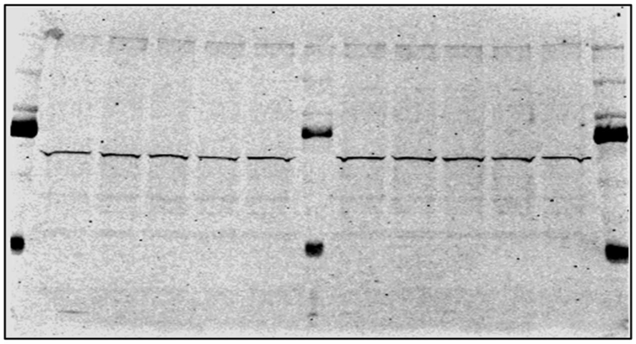

Western blots corresponding with Figure 5

Fig. 5d – NFAT1 blot
(Lower exposure)
Left six lanes: cytoplasmic fraction
Right six lanes: nuclear fraction

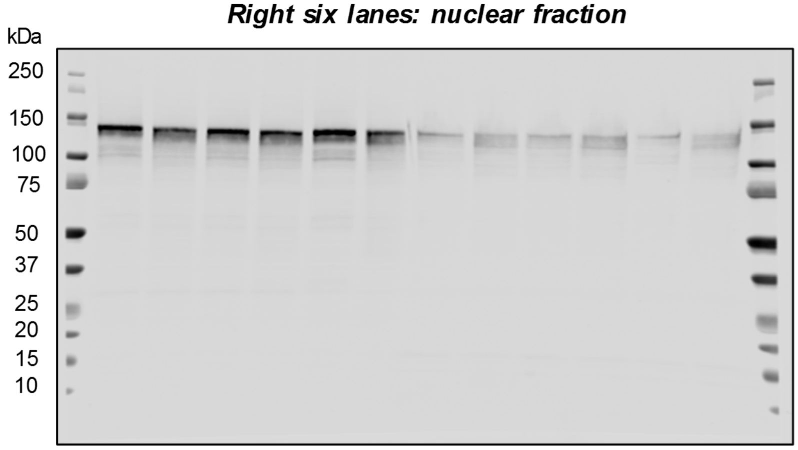

Fig. 5d – NFAT1 blot
(Higher exposure)
Left six lanes: cytoplasmic fraction
Right six lanes: nuclear fraction

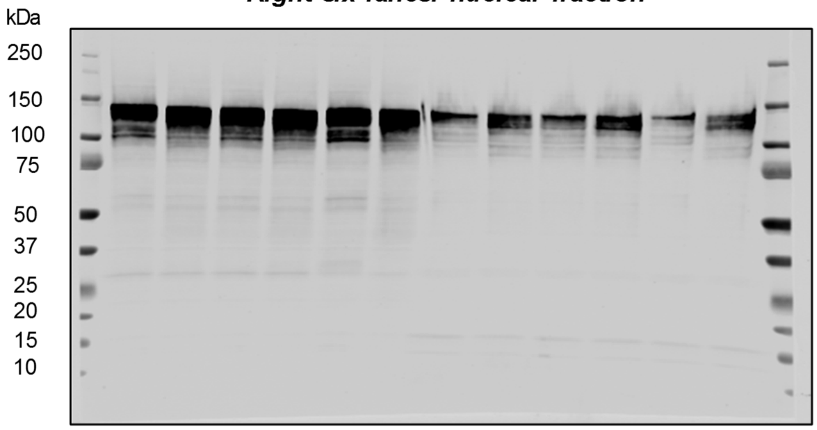

Fig. 5d – Beta tubulin and Histone H3 blot
Left six lanes: cytoplasmic fraction
Right six lanes: nuclear fraction

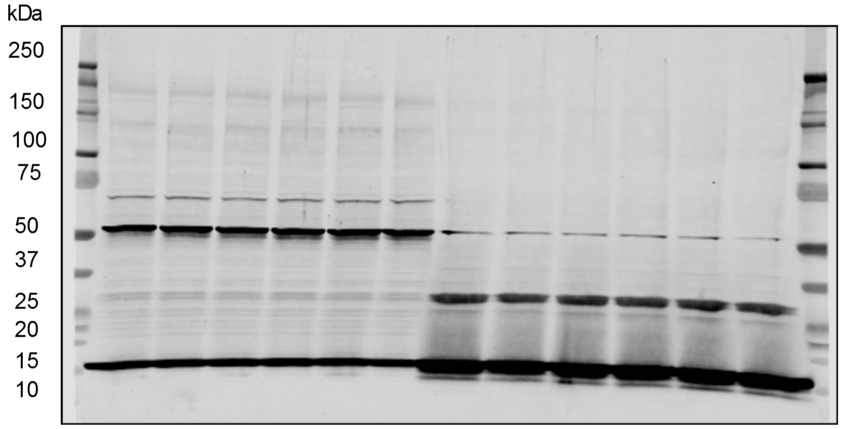

Western blots corresponding with Figure 7

<u>Fig. 7b</u> – CD22, SHP-1, Gal-9 blot

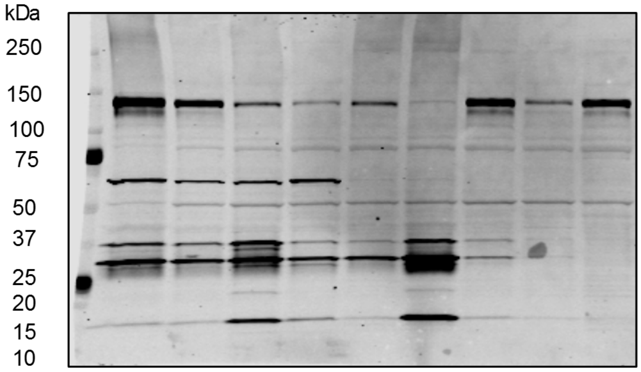

<u>Fig. 7b</u> – Alpha-tubulin blot

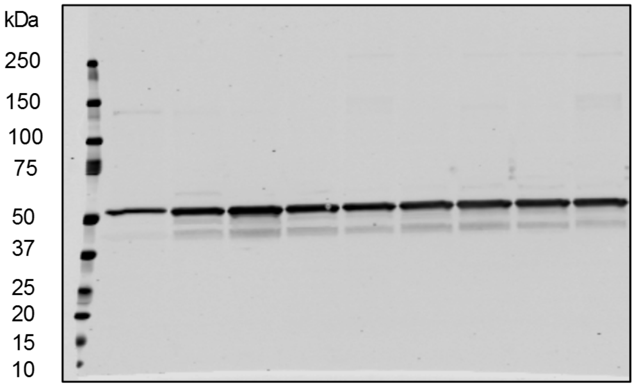

Western blots corresponding with Supplementary Fig. 5

Supp. Fig 5d
Streptavidin blot of biotinylated Gal-9 IP lysates

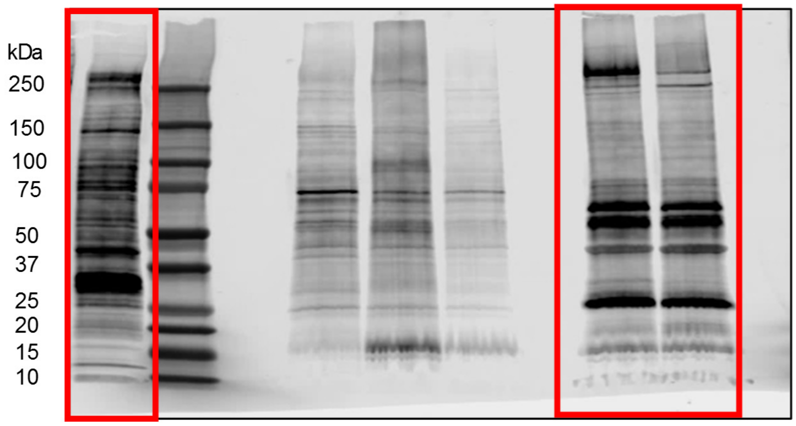

Supp. Fig 5e
Gal-9 IP, blot CD45

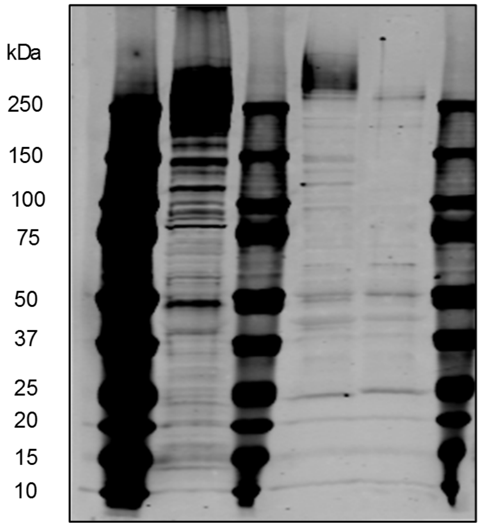

Supp. Fig 5e
CD45 IP, blot Gal-9

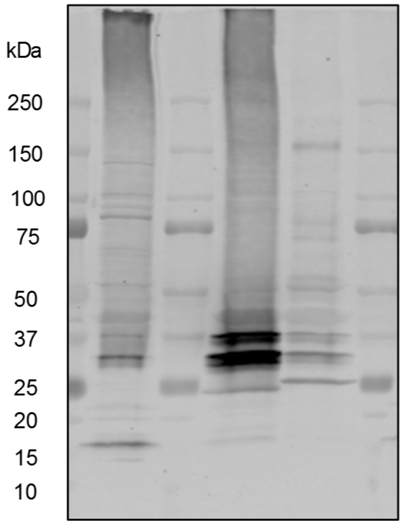

Western blots corresponding with Supplementary Fig. 6 (1 of 2)

<u>**Supp. Fig 6a – pTyr**</u>
4G10 blot
Left lanes: No IgM stim
Right lanes: IgM stim

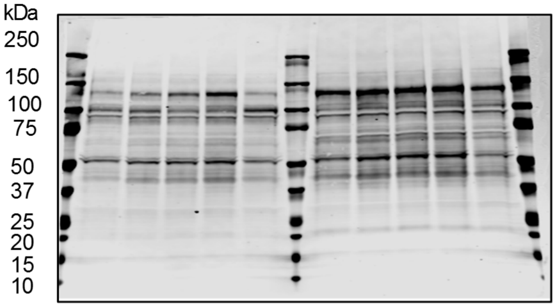

<u>**Supp. Fig 6a – pTyr**</u>
Beta actin blot
Left lanes: No IgM stim
Right lanes: IgM stim

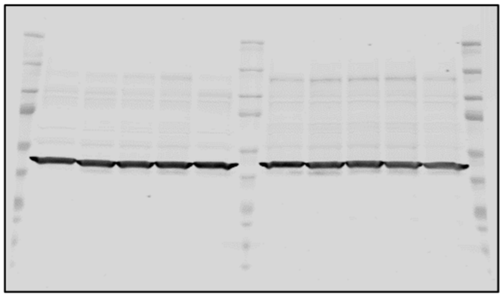

<u>**Supp. Fig 6c – pCD79a**</u>
pCD79a Y182 blot
(also blotted with pPLCγII and pSy k)

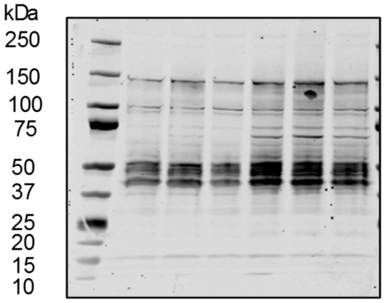

<u>**Supp. Fig 6c – pCD79a**</u>
Total CD79a blot
(also blotted with total pPLCγII and total Sy k)

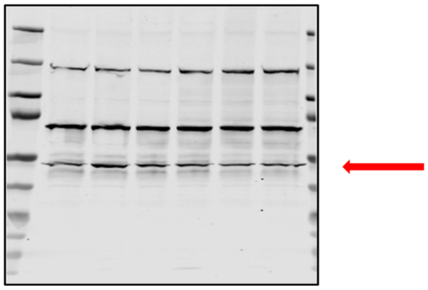

<u>**Supp. Fig 6c – pCD79a**</u>
pSyk Y525/Y526
(also blotted with pPLCγII and pCD79a)

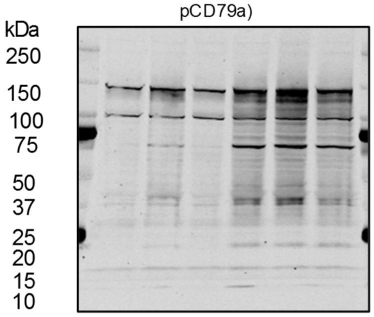

<u>**Supp. Fig 6c – pCD79a**</u>
Total Syk
(also blotted with total pPLCγII and total CD79a)

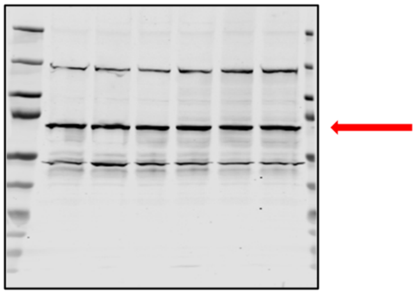

<u>**Supp. Fig 6c – pBLNK**</u>
pBLNK Y96
(also blotted with another antibody at 145kDa)

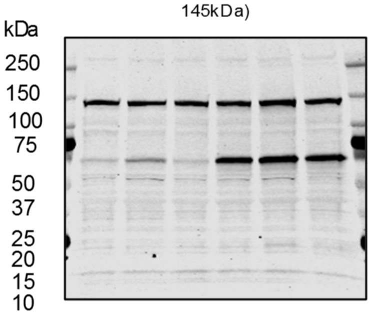

<u>**Supp. Fig 6c – pBLNK**</u>
Total BLNK
(also blotted with other total antibodies at ~100 and 145kDa)

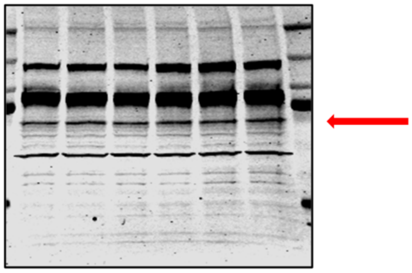

Western blots corresponding with Supplementary Fig. 6 (2 of 2)

Supp. Fig 6c – pPLCγII
pPLCγII Y759
(also blotted with pCD79a)

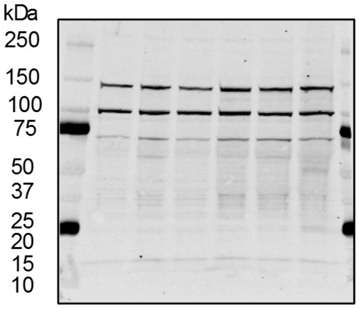

Supp. Fig 6c – pPLCγII
Total PLCγII
(also blotted with total CD79a)

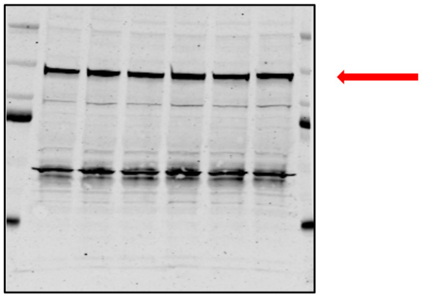

Supp. Fig 6c – pAkt and pErk
pAkt S473 ; pErk 1/2 T202 / Y204
(co-blotted)

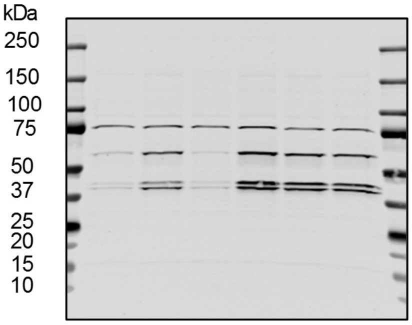

Supp. Fig 6c – pAkt and pErk
Total Akt and Total Erk
(co-blotted)

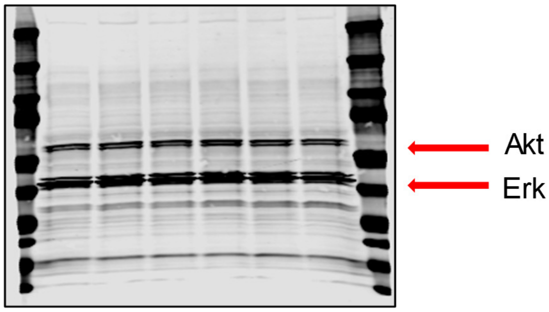

Supp. Fig 6c – pJNK
pJNK (T183/Y185)

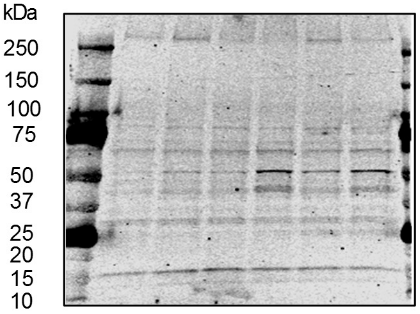

Supp. Fig 6c – pJNK
Total JNK

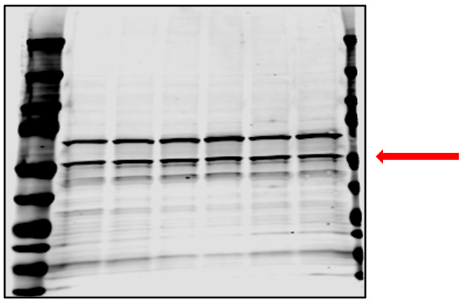

Supp. Fig 6c – pNFκB p65
pNFκB p65 (S536)

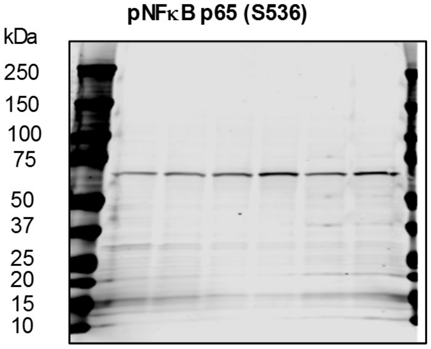

Supp. Fig 6c – pNFκB p65
Total NFκB p65

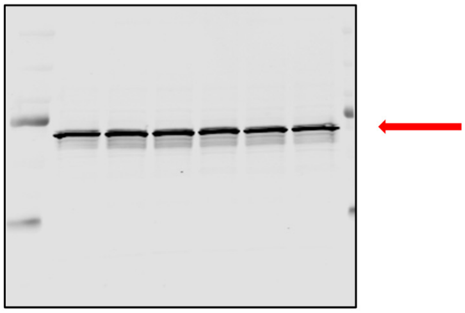

Western blots corresponding with Supplementary Fig. 8

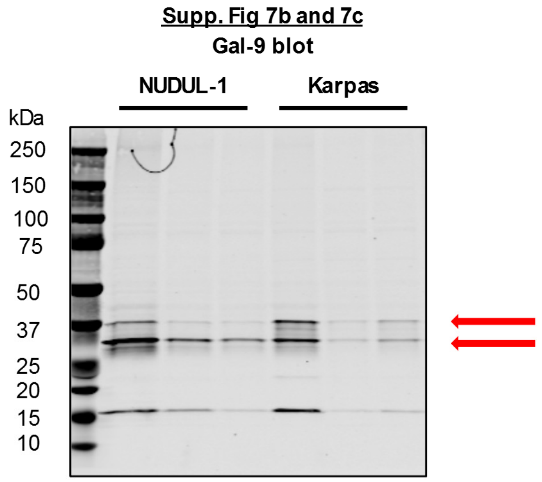

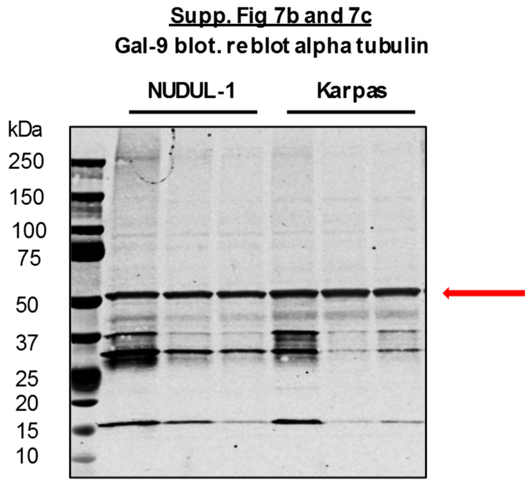

Supplementary Figure 9: Full Western blots from this study. Full-length Western blots of data presented in the main and supplementary figures of this study. Red boxes indicate lanes that are presented in cropped blots. Where blots have been re-probed with additional antibodies, the red arrow points to the band(s) corresponding with the indicated protein.

Supplementary Table 1. Oligonucleotide sequences.

Gene	Forward Sequence (5'-3')	Reverse Sequence (5'-3')	Comments
GCNT2, human	ATGCATCCTGGACTGGAAAC	AGCAAACAGGCTTGGTGAAT	qRT-PCR, 60º
LGALS9, human	CATCCAAGTCCATCCTCCTG	GGAGTTGTCGATCTGGGTGT	qRT-PCR, 60º
VCP, human	AGGATGATCCAGTGCCTGAG	GGAATCTGAAGCTGCCAAAG	qRT-PCR, 60º
Non-target control shRNA	CAACAAGATGAAGAGCACCAA	-	-
Human *GCNT2* shRNA #1	GCTCACCTCTATATTAGTTTA	-	-
Human *GCNT2* shRNA #2	GCTAACAAGTTTGAGCTTAAT	-	-
Human *LGALS9* shRNA #1	TGGTCAGCACCTGTTTGAATA	-	-
Human *LGALS9* shRNA #2	CCCTCCTCTCTGACCTTTAAC	-	-

Supplementary table 2: Antibodies and reagents used in this study.

**Lectins and glycobiology reagents**						
**Target**	**Conjugate**	**Clone**	**Source**	**Catalog number**	**Concentration / Dilution**	**Incubation time**
Recombinant human Galectin-1, *E.Coli* derived	-	-	PeproTech	450-39	50μg/mL (FACS)	45min, ice
Recombinant human Galectin-3, *E.Coli* derived	-	-	PeproTech	450-38	10μg/mL (FACS)	45min, ice
Recombinant human Galectin-9, *E.Coli* derived	-	-	R&D	2045-GA	1μg/mL (FACS); 0.5-4μg/mL (Functional assays)	45min, ice
Phaseolus vulgaris Leucoagglutinin (PHA-L)	Biotin	-	Vector	B-1115	0.1μg/mL (FACS)	45min, ice
Lycopersicon Esculentum Agglutinin (LEA)	Biotin	-	Vector	B-1175	0.1μg/mL (FACS)	45min, ice
Solanum Tuberosum Agglutinin (STA)	Biotin	-	Vector	B-1165	0.1μg/mL (FACS)	45min, ice
Sambucus Nigra Agglutinin (SNA)	Biotin	-	Vector	B-1305	0.25μg/mL (FACS)	45min, ice

Erythrina Cristagalli Agglutinin (ECA)	Biotin	-	Vector	B-1145	0.04μg/mL (FACS)	45min, ice
Arthrobacter ureafaciens sialidase	-	-	Vector	10269611001	125mU / Ml	1hr, RT
**Flow cytometry antibodies and staining reagents**						
**Target**	**Conjugate**	**Clone**	**Source**	**Catalog number**	**Concentration / Dilution**	**Incubation time**
CD3	APC-Cy7	HIT3a	Biolegend	300318	1:100 (FACS)	45min, ice
CD14	APC-Cy7	HCD14	BIolegend	325620	1:160 (FACS)	45min, ice
CD19	PerCP	HIB19	Biolegend	302228	1:40 (FACS)	45min, ice
CD19	APC	HIB19	Biolegend	302212	1:100 (FACS)	45min, ice
CD19	APC-Cy7	HIB19	Biolegend	302218	1:40 (FACS)	45min, ice
CD22	PE	S-HCL-1	Biolegend	363504	1:25 (FACS)	45min, ice
CD27	PE-Cy7	LG.3A10	Biolegend	124216	1:160 (FACS)	45min, ice
CD38	PE	HB-7	Biolegend	356604	1:160 (FACS)	45min, ice
CD44	APC	IM7	Biolegend	103012	1:400 (FACS)	45min, ice
CD45	APC	HI30	Biolegend	304012	1:25 (FACS)	45min, ice
CD86	PE	IT2.2	Biolegend	305406	1:80 (FACS)	45min, ice
Donkey anti-goat IgG (H+L)	APC	Poly	R&D	F0108	1:10 (FACS)	45min, ice
Ki-67	PE	Ki-67	Biolegend	350504	1:40 (FACS)	45min, ice
Galectin-1	-	Poly	R&D	AF1152	2.5 μg/mL (FACS)	45min, ice
Galectin-3	AlexaFluor 647	M3/38	Biolegend	125408	1 μg/mL (FACS)	45min, ice
Galectin-9	APC	9M1-3	Biolegend	348908	1:50 (FACS)	45min, ice
Human Fc block	-	-	Biolegend	422301	1:40 (FACS)	45min, ice
IgD	FITC	IA6-2	BIolegend	348206	1:200 (FACS)	45min, ice
IgM	APC	MHM-88	BIolegend	314510	1:50 (FACS)	45min, ice
Zombie NIR Fixable Viability Kit	-	-	Biolegend	423106	1:1600 (FACS)	45min, ice
**Magnetic sorting antibodies and reagents**						
**Target**	**Conjugate**	**Clone**	**Source**	**Catalog number**	**Concentration / Dilution**	**Incubation time**
B cell isolation kit II, human	-	-	Miltenyi	130-091-151	Manufact. guidelines	Manufact. guidelines
Anti-FITC microbeads	-	-	Miltenyi	130-048-701	Manufact. guidelines	Manufact. guidelines
CD10	Biotin	97C5	Miltenyi	130-093-451	Manufact. guidelines	Manufact. guidelines
CD27	Biotin	LGA.3A10	Biolegend	124206	25μg/mL (MACS)	10min, ice
CD77	FITC	5B5	Biolegend	357104	10μg/mL (MACS)	10min, ice
**Western blot and immunoprecipitation reagents**						
**Target**	**Conjugate**	**Clone**	**Source**	**Catalog number**	**Concentration / Dilution**	**Incubation time**
Akt, pan	-	40D4	CST	2920	1:1000 (WB)	1hr, RT
β-tubulin	-	Poly	Santa Cruz	sc-9104	1:200 (WB)	1hr, RT
BLNK	-	D3P2H	CST	36438	1:1000 (WB)	1hr, RT
CD22	-	219902	R&D	MAB19681	1:3000 (WB)	1hr, RT

CD45 (WB)	-	D9M8I	CST	13917S	1:1000 (WB)	O/N, 4C
CD45 (IP)	-	F10-89-4	BioRad	MCA87GA	20μg/mL (IP)	O/N, 4C
CD79a	-	Poly	CST	3351	1:1000 (WB)	1hr, RT
Erk1/Erk2	-	216703	R&D	MAB1576	1:500 (WB)	1hr, RT
Galectin-9 (for IP)	-	9M1-3	Biolegend	348902	13.3μg/mL (IP)	O/N, 4C
Galectin-9 (for WB)	-	Poly	R&D	AF2045	1μg/mL (WB)	O/N, 4C
Histone H3	-	D1H2	CST	4499	1:2000 (WB)	1hr, RT
Lyn	-	LYN-01	Biolegend	628102	1:500 (WB)	1hr, RT
Ms IgG1 Isotype control (IP)	-	MOPC-21	Biolegend	400102	13.3mg/mL (IP)	O/N, 4C
Ms IgG2a Isotype control (IP)	-	-	BD	557353	20mg/mL (IP)	O/N, 4C
NFAT1	-	D43B1	CST	5861	1:1000 (WB)	1hr, RT
NFkB p65	-	L8F6	CST	6956	1:1000 (WB)	1hr, RT
Phopsho-Akt (S473)	-	D9E	CST	4060	1:1000 (WB)	O/N, 4C
Phospho-BLNK (Y96)	-	Poly	CST	3601	1:1000 (WB)	O/N, 4C
Phospho-CD22 (Y842)	-	Y507	Abcam	ab32355	1:2000 (WB)	O/N, 4C
Phospho-CD79a (Y182)	-	Poly	CST	5173	1:1000 (WB)	O/N, 4C
Phospho-Lyn (Y507)	-	Poly	CST	2731	1:1000 (WB)	O/N, 4C
Phospho-NFkB p65 (S536)	-	93H1	CST	3033	1:2000 (WB)	O/N, 4C
Phospho-p44/42 MAPK (Erk1/2) (T202/Y204)	-	D13.14.4 E	CST	4370	1:2000 (WB)	O/N, 4C
Phospho-SAPK / JNK (T183/Y185)	-	G9	CST	9255	1:500 (WB)	O/N, 4C
Phospho-SHP-1 (Y564)	-	D11G5	CST	8849	1:1000 (WB)	O/N, 4C
Phospho-Syk (Y525/526)	-	C87C1	CST	2710	1:1000 (WB)	O/N, 4C
Phospho-tyrosine	-	4G10	Millipore Sigma	05-321	1μg/mL (WB)	O/N, 4C
SAPK/JNK	-	Poly	CST	9252	1:1000 (WB)	1hr, RT
SHP-1	-	C14H6	CST	3759	1:1000 (WB)	1hr, RT
Syk	-	D3Z1E	CST	13198	1:1000 (WB)	1hr, RT
Donkey anti-Goat IgG (H+L)	IRDye® 800CW	Poly	Li-Cor	926-32214	1:20,000 (WB)	30min, RT
Goat anti-Rabbit IgG (H+L)	IRDye® 800CW	Poly	Li-Cor	926-32211	1:20,000 (WB)	30min, RT
Goat anti-Mouse IgG (H+L)	IRDye® 800CW	Poly	Li-Cor	926-32210	1:20,000 (WB)	30min, RT
Goat anti-Rabbit IgG (H+L)	IRDye® 680LT	Poly	Li-Cor	926-68023	1:20,000 (WB)	30min, RT

Goat anti-Mouse IgG (H+L)	IRDye® 680RD	Poly	Li-Cor	926-68070	1:20,000 (WB)	30min, RT
Streptavidin	IRDye® 800CW	-	Li-Cor	926-32230	1:10,000 (WB)	30min, RT
NewBlot IR Stripping Buffer	-	-	Li-Cor	928-40028	Manufact. guidelines	Manufact. guidelines
**Immunofluorescence microscopy antibodies and reagents**						
**Target**	**Conjugate**	**Clone**	**Source**	**Catalog number**	**Concentration / Dilution**	**Incubation time**
NFAT1	-	D43B1	CST	5861	1:50 (IF)	O/N, 4C
CD45	AlexaFluor 488	F10-89-4	Abcam	ab197730	1:20 (IF)	30min, RT
Donkey anti-rabbit IgG	AlexaFluor 555	Poly	Biolegend	406412	1:250 (IF)	1hr, RT
Poly-L-lysine coated slides	-	-	Ibidi	80604	-	-
Poly-L-lysine coated round 12mm coverslips	-	-	Corning	354085	-	-
Prolong Gold with DAPI	-	-	CST	8961	-	-
**Immunohistochemistry antibodies and reagents**						
**Target**	**Conjugate**	**Clone**	**Source**	**Catalog number**	**Concentration / Dilution**	**Incubation time**
Galectin-9	-	9S2-1	Biolegend	650702	10μg/mL (IHC)	30min, RT
PAX5	-	D7H5X	CST	12709	1:50 (IHC)	30min, RT
Ms IgG1 Isotype control	-	-	Biolegend	400102	10μg/mL (IHC)	30min, RT
Solanum Tuberosum Agglutinin (STA)	Biotin	-	Vector Laboratories	B-1165	2μg/mL (IHC)	30min, RT
**Stimulation and cell culture reagents**						
**Target**	**Conjugate**	**Clone**	**Source**	**Catalog number**	**Concentration / Dilution**	**Incubation time**
AffiniPure F(ab')$_2$ Fragment Goat anti-Human IgM	-	Poly	Jackson ImmunoRes.	109-006-129	10μg/mL (Activation); 15μg/mL (Signaling); 20μg/mL (Ca2+ flux)	-
AffiniPure F(ab')$_2$ Fragment Donkey Anti-human IgG (H+L)	-	Poly	Jackson ImmunoRes.	709-006-149	20μg/mL (Ca2+ flux)	-
Goat F(ab')$_2$ anti-human IgD-UNLB	-	Poly	Southern Bio	2032-01	10μg/mL (Activation); 20μg/mL (Ca2+ flux)	-
Goat F(ab')$_2$ anti-human Kappa-UNLB	-	Poly	Southern Bio	2062-01	10μg/mL (Activation); 20μg/mL (Ca2+ flux)	-

Goat F(ab')$_2$ anti-human Lambda-UNLB	-	Poly	Southern Bio	2072-01	10μg/mL (Activation); 20μg/mL (Ca2+ flux)	-
CpG Oligonucleotides (ODN2006)	-	-	Invivogen	tlrl-2006	0.1 μM	-
Recombinant human CD40L-Trimer	-	-	Gift (G. Freeman (DFCI/Harvard)	-	1μg/mL	-
Recombinant human IL-2	-	-	Biolegend	589102	10ng/mL	-
Recombinant human IL-4	-	-	Biolegend	574002	20ng/mL	-
Recombinant human IL-10	-	-	Biolegend	571002	20ng/mL	-
Recombinant human IL-21	-	-	Biolegend	571202	50ng/mL	-
